# Lipid-encapsulated siRNA for hepatocyte-directed treatment of advanced liver disease

**DOI:** 10.1038/s41419-020-2571-4

**Published:** 2020-05-11

**Authors:** Marius Maximilian Woitok, Miguel Eugenio Zoubek, Dennis Doleschel, Matthias Bartneck, Mohamed Ramadan Mohamed, Fabian Kießling, Wiltrud Lederle, Christian Trautwein, Francisco Javier Cubero

**Affiliations:** 1 Department of Internal Medicine III, University Hospital RWTH Aachen, Aachen, Germany; 2 Department of Toxicology and Pharmacology, School of Nutrition, Toxicology and Metabolism (NUTRIM), Maastricht University Medical Centre Maastricht University, Maastricht, The Netherlands; 3 Institute for Experimental and Molecular Imaging, University Hospital RWTH Aachen, Aachen, Germany; 4 Department of Therapeutic Chemistry, National Research Centre, 12622 Cairo, Egypt; 5 Department of Immunology, Ophthalmology and ENT, Complutense University School of Medicine, Madrid, Spain; 6 12 de Octubre Health Research Institute (imas12), Madrid, Spain

**Keywords:** RNAi, Chronic inflammation

## Abstract

Lipid-based RNA nanocarriers have been recently accepted as a novel therapeutic option in humans, thus increasing the therapeutic options for patients. Tailored nanomedicines will enable to treat chronic liver disease (CLD) and end-stage liver cancer, disorders with high mortality and few treatment options. Here, we investigated the curative potential of gene therapy of a key molecule in CLD, the c-Jun N-terminal kinase-2 (*Jnk2*). Delivery to hepatocytes was achieved using a lipid-based clinically employable siRNA formulation that includes a cationic aminolipid to knockdown *Jnk2* (named *siJnk2*). After assessing the therapeutic potential of *siJnk2* treatment, non-invasive imaging demonstrated reduced apoptotic cell death and improved hepatocarcinogenesis was evidenced by improved liver parenchyma as well as ameliorated markers of hepatic damage, reduced fibrogenesis in 1-year-old mice. Strikingly, chronic *siJnk2* treatment reduced premalignant nodules, indicative of tumor initiation. Furthermore, *siJnk2* treatment led to a significant activation of the immune cell compartment. In conclusion, *Jnk2* knockdown in hepatocytes ameliorated hepatitis, fibrogenesis, and initiation of hepatocellular carcinoma (HCC), and hence might be a suitable therapeutic option, to define novel molecular targets for precision medicine in CLD.

## Introduction

Viral-based delivery systems for nucleic acids have demonstrated high levels of transfection efficiency and highly potent delivery^1^, but are hampered by their known side effects such as immunogenicity and therefore have not made it into clinical practice^2^. While RNA delivery has been an obstacle for decades, lipid-based delivery systems using cationic or ionizable lipids have recently been understood at greater depth^2–5^.

In recent years, significant progress has been made to overcome some of the obstacles associated with in vivo delivery of siRNA, and lipid nanoparticles (LNPs) are very promising tools. LNPs have been specifically tested for hepatocyte delivery of siRNA molecules, since the liver is a well-perfused organ with a fenestrated endothelium. On the other hand, LNPs are known to interact with serum proteins, exchanging components and acquiring proteins in circulation that can potentially direct LNPs to specific cell types^6^. It is well-known that siRNA-loaded LNP absorb Apolipoprotein E (ApoE) on their surface, enhancing uptake into hepatoma cells and primary hepatocytes^6,7^. ApoE binds to the low-density lipoprotein receptor (LDLR), which is strongly expressed on the outer membrane of hepatocytes. In short, LNPs behave as neutral liposomes in circulation, acquiring ApoE and delivering siRNA to hepatocytes in a targeted manner.

This mechanism of hepatocyte uptake of LNP via the LDLR route is the basis of the first FDA-approved siRNA-based drug, Patisiran, a medication for the treatment of polyneuropathy in people with hereditary transthyretin-mediated amyloidiosis^8^. Patisiran is an siRNA, which targets hepatocytes and prevents the production of proteins, which do not fold correctly and thus accumulate in different parts of the body, including the central nervous system^9^.

Liver cancer represents globally the second most common cause of cancer-related death and its incidence is rising dramatically^10^. Hepatocellular carcinoma (HCC) is the most common type of liver cancer, arising almost exclusively from inflammatory processes, in which hepatocytes signaling play a pivotal role^11^. Unresolved, chronic inflammation potentially leads to fibrosis, cirrhosis and supports HCC development. Both liver cirrhosis and HCC, in turn, can lead to complete liver failure and consequently, without liver transplantation, to the death of the patient. Previous publications of our group demonstrated that deletion of the regulatory subunit IKKγ (NEMO) in murine hepatocytes causes spontaneous HCC development preceded by chronic liver disease mimicking human non-alcoholic steatohepatitis (NASH)^12,13^.

Lack of NEMO expression specifically in hepatocytes leads to complete NF-κB inactivation and, in turn, to sustained JNK activation and reactive oxygen species (ROS) formation promoting cellular death^12^. Impaired NEMO expression in HCC patients was correlated with poor survival^14^. In recent publications, we have explored the specific roles of the *Jnk* genes during liver disease^13,15^.

Whereas *Jnk1* plays a pro-apoptotic and pro-tumorigenic function, *Jnk2* seems to modulate fibrogenesis. Lately, a protective role for *Jnk2* was shown against Ibuprofen-mediated acute liver failure (ALF)^15^. However, many aspects of the function of *Jnk2*, particularly in HCC, remain currently unknown.

The use of genetically modified mice with gain or loss-of-function of individual genes is the gold standard to study the involvement of proteins biological processes in vivo^16^. In this study, we first studied the functions of *Jnk2* in liver cancer using a model that reflects the situation in patients with end-stage HCC^12^. The experimental model of chronic liver disease (CLD) and end-stage carcinogenesis is based on the NEMO^∆Hepa^ mice, which bear a gene promoter-specific deletion (Albumin-Cre/loxP-mediated) of NEMO specifically in hepatocytes. Next, NEMO^∆Hepa^ mice were compared to mice also deficient in *Jnk2* in hepatocytes, called NEMO/JNK2^∆Hepa^ mice. We evaluated the therapeutic potential of targeting hepatocytes in HCC, using LNP-based delivery of small interfering RNA (siRNA) directed against *Jnk2* (*siJnk2-*LNP). The *siJnk2-*LNP were carefully optimized by exploring 12 different siRNA sequences. The LNP were formulated in a clinically applicable fashion using a cationic aminolipid. The formulation efficiently accumulates in the liver, and most efficiently silences *Jnk2* in hepatocytes, which are main drivers of the inflammatory circuit that underlies HCC. Inhibition of *Jnk2* ultimately reduced carcinogenesis in a model of advanced liver cancer.

## Results

### Design and validation of siRNA sequence for *Jnk2* inhibition

Nanomedicines improve clinical options for many types of liver diseases including inflammation, fibrosis, or cancer. Cell-type-specific targeting has the potential to significantly reduce side effects and improve efficacy^17^. Lipid-based formulations are enriching clinical routine and belong to the most successful tumor therapeutics. However, the options for liver cancer are still limited and novel treatment options are urgently required. In order to explore a new concept for the treatment of liver cancer, we comprehensively studied the functions of *Jnk2* in a genetically determined HCC mouse model, the NEMO^∆Hepa^ mouse. In the next step, we discovered the usability of siRNA molecules targeting *Jnk2* siRNA, which were formulated in a clinically applicable LNP.

**Fig. 1:**
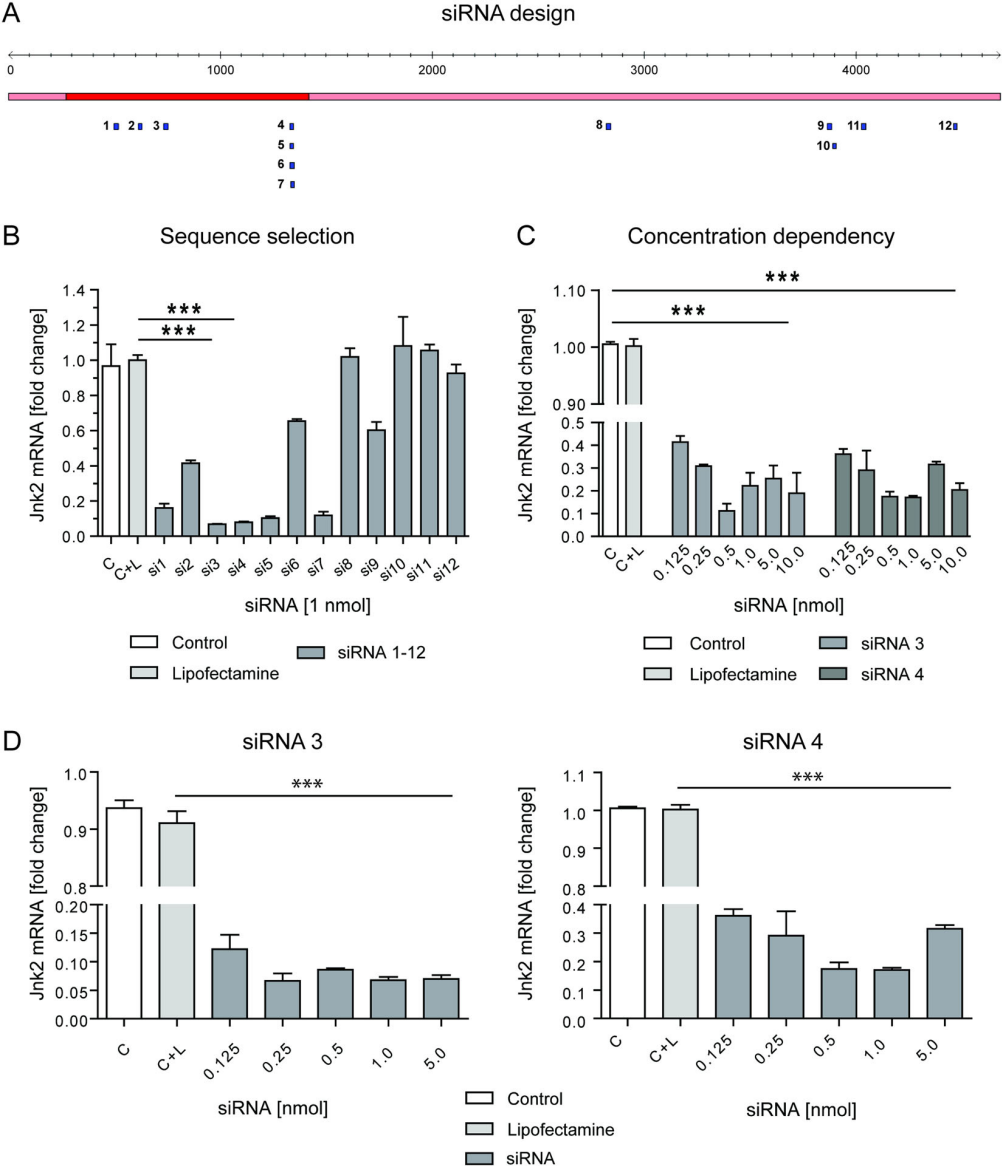
Design and validation of siRNA sequence for *Jnk2* inhibition. **a** Bioinformatics-based sequence design for the inhibiting *Jnk2* mRNA resulted in 12 different siRNA sets. The different binding regions of each siRNA set is shown. **b** Selection of the most efficient siRNA sets targeting *Jnk2* in cell-culture experiments. Hepa 1–6 were treated with either medium only, or transfected with Lipofectamine or one out of 12 different computer-defined Jnk2-siRNAs [1 nmol] for 24 h and the *Jnk2* mRNA expression was quantified by using qRT-PCR. Results are expressed as fold increase. **c** Hepa 1–6 cells were treated with medium only or transfected with Lipofectamine or *siJnk2* (which corresponded to siRNA sets 3 or 4) in a concentration ranging from 0.125–10 nmol for 24 h and subsequently *Jnk2* mRNA levels were determined by using qRT-PCR. Results are expressed as fold increase. **d** Primary murine WT hepatocytes received either medium only, or were transfected with Lipofectamine or *siJnk2* sets 3 or 4 in a concentration ranging from 0.125–5 nmol for 24 h. The mRNA levels of *Jnk2* were quantified by using qRT-PCR and results are shown as fold increase. Results are expressed as mean ± S.E.M. ***P < 0.001.

The first step to characterize the role of JNK2 in CLD was to select the most potent siRNA sequence for the knockdown of *Jnk2*. In order to achieve the highest efficiency in blocking *Jnk2*, we first designed 12 different sequences for the *siJnk2-*LNP that target the mRNA via different binding regions (Fig. 1a). Detailed information on the sequences is provided in the supplementary information (Supplementary Table 1). To identify the most efficient siRNA molecules to knockdown *Jnk2*, we subsequently explored their efficiency in different liver cell types using a commercial delivery system, so-called lipoplexes. We first transfected Hepa 1–6 cells, a mouse hepatoma cell line, with a panel of 12 different computer-defined siRNAs for 24 h and tested *Jnk2* mRNA expression (Fig. 1b). As the protein Luciferase is not expressed in the liver, a siRNA targeting Luciferase was used as a negative control.

This screening approach defined *siRNA-Jnk2* set 3 and 4 to downregulate *Jnk2* mRNA to less than 10% of the original level. Dose finding experiments identified a concentration of 0.5 nmol as the most potent siRNA application (Fig. 1c). Efficacy was further confirmed in primary isolated wild-type (WT) hepatocytes. Both siRNA molecules significantly downregulated *Jnk2* mRNA expression levels in a concentration ranging from 0.125 to 5.0 nmol 24 h after transfection. The most potent concentration of *Jnk2* knockdown was found at a concentration of 0.25 nmol and 0.5 nmol for the siRNA set 3 and 4, respectively (Fig. 1d).

### Genetic deletion of *Jnk2* reduces liver cancer at advanced stages

In this first part of our study, bioinformatics analysis identified highly potent siRNA sequences that efficiently knockdown *Jnk2* in hepatocytes. Comprehensive data providing a key role for *Jnk2* in acute liver failure (ALF) from our group was recently obtained^15^. In this earlier study, *siJnk2-*LNP treatment prior to ibuprofen administration caused a dramatic increase in hepatic damage and cell death. However, due to stage-dependent properties of the tumor microenvironment, targeting *Jnk2* in advanced CLD might nevertheless represent a therapeutic option.

**Fig. 2:**
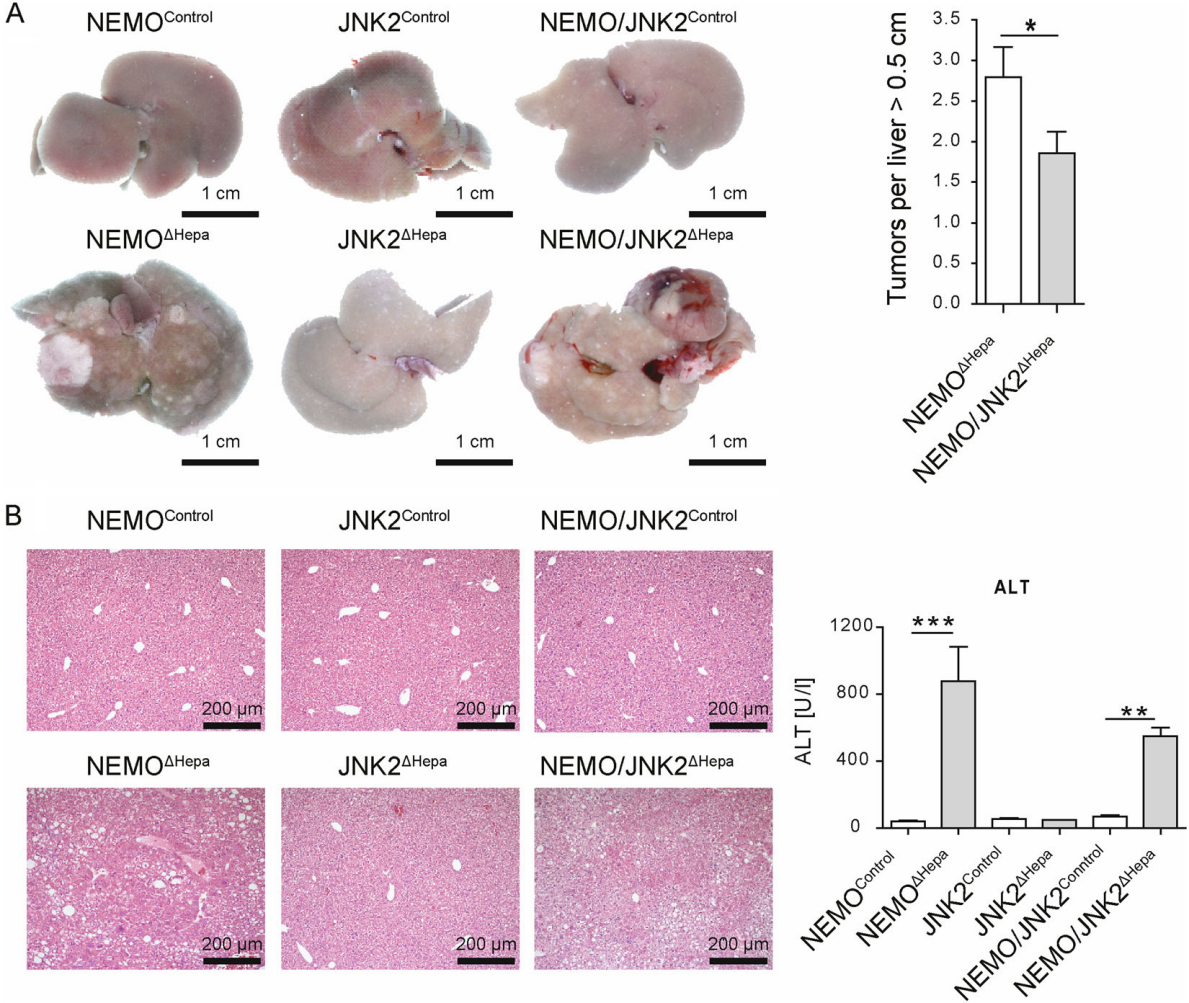
Genetic deletion of *Jnk2* reduces liver cancer at advanced stages. NEMO^ΔHepa^, JNK2^ΔHepa^, and NEMO/JNK2^ΔHepa^ mice and their respective control animals (NEMO^Control^, JNK2^Control^, and NEMO/JNK2^Control^ were generated. Mice were sacrificed at the age of 52 weeks. Livers were extracted and analyzed macros- and microscopically for phenotypic characterization. **a** Macroscopic images of livers of 1-year-old control mice (upper images) and their corresponding knockout mice NEMO^ΔHepa^, JNK2^ΔHepa^, and NEMO/JNK2^ΔHepa^ (lower images), scale bars: 1 cm. Number of tumor nodules ≥0.5 cm in diameter were quantified in livers of NEMO^ΔHepa^ and NEMO/JNK2^ΔHepa^ animals at the age of 52 weeks (right). **b** Representative histological liver sections of the identical control animals (upper images) and the knockout mice (upper images) at the age of 52 weeks were stained with hematoxylin and eosin (left). Serum liver ALT (right) of 52-week-old animals is displayed in U/l (*n* = 11–13). (Scale bars: 100 μm).

In the next step, we therefore studied the impact of JNK2 protein on the formation of liver tumors. Previous studies suggested a major role of the NF-κB pathway and JNK activation for HCC development^12,13^. Thus, we generated double-knockout mice with hepatocyte-specific deletion of JNK2 and NEMO^ΔHepa^ and subsequently examined liver cancer progression in NEMO^ΔHepa^, NEMO/JNK2^ΔHepa^ mice, and their corresponding littermate controls (floxed mice). First, the impact of *Jnk2* deletion on 1-year-old NEMO^ΔHepa^ mice was evaluated. Macroscopic analysis of 1-year-old NEMO/JNK2^ΔHepa^ elicited a significant reduction in the tumor number compared with NEMO^ΔHepa^, while controls did not display any evidence of tumorigenesis (Fig. 2a, left and right panels). H&E staining confirmed the architecture of liver parenchyma of NEMO/JNK2^ΔHepa^ characterized by decreased dysplasia, inflammation, and steatosis, compared with NEMO^ΔHepa^ mice (Fig. 2b). In-line with these results, markers of liver damage in NEMO/JNK2^ΔHepa^ manifested an overall improvement of liver disease. Aspartate aminotransferase (AST), glutamate dehydrogenase (GLDH), and alkaline phosphatase (AP) were significantly decreased in NEMO/JNK2^ΔHepa^ compared with 1-year old NEMO^ΔHepa^ animals (Supplementary Fig. 1a–c). In addition, a clear tendency towards reduced alanine aminotransferase (ALT) was also found in 1-year old NEMO/JNK2^ΔHepa^ mice (Fig. 2b). Moreover, hepatocytic JNK2 deletion led to overall improved hepatic fibrogenesis as observed by Sirius red (SR) staining and quantification (Supplementary Fig. 2d). Altogether, these results indicate that deletion of JNK2 specifically in hepatocytes in experimental CLD mouse model might be a suitable treatment to reduce end-stage HCC formation.

### Lipid-based nanoparticles for organ and cell-type-specific delivery of siRNA molecules

**Fig. 3:**
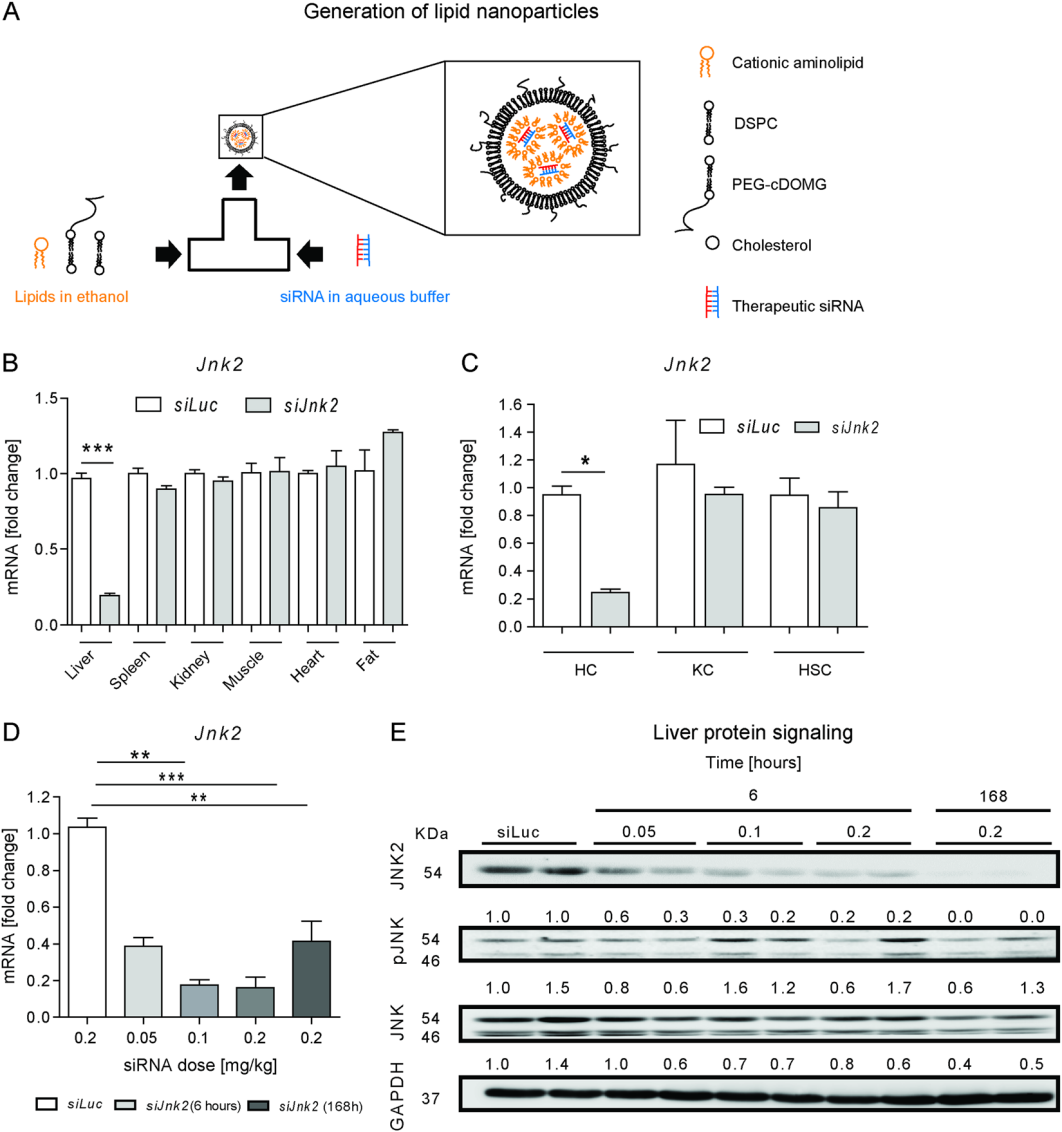
Lipid-based nanoparticles for organ and cell-type-specific delivery of siRNA molecules. **a** Scheme of T-junction-based production of lipid-based nanoparticles for siRNA delivery, which are composed of a lipid mixture containing the aminolipid KL-52, 1,2-distearoyl-3-phosphatidylcholine (DSPC), α-[3’-(1,2-dimyristoyl-3-propanoxy)-carboxamide-propyl]-ω-methoxy-polyoxyethylene (PEG-c-DOMG), and cholesterol dissolved in ethanol and of a therapeutic siRNA in aqueous buffer. **b** C57BL6J mice aged 7–8 weeks were treated with a single dose of luciferase (*siLuc*) or *siJnk2-*LNP in vivo with a concentration of 0.2 mg/kg BW for 6 h (*n* = 3). *Jnk2* mRNA expression was determined in isolated liver, spleen, kidney, muscle, heart, and fat tissue. **c** *Jnk2* mRNA levels were analyzed in hepatocytes, KC and HSC isolated from livers of WT mice that were treated with either *siLuc* or *siJnk2*-LNP. **d** *Jnk2* mRNA expression in whole liver extracts in doses ranging from 0.05 to 0.2 mg/kg after 6 or 168 h at the indicated doses. Quantitative RT-PCR results were displayed as fold induction. **e** Protein levels of JNK2, pJNK, and JNK were analyzed by immunoblotting of whole liver extracts at siRNA doses ranging from 0.05 to 0.2 mg/kg for 6 or 168 h. GAPDH was used as loading control. Ratio: Normalization of each protein level relative to GAPDH was determined by densitometry. Results are expressed as means ± S.E.M. *P < 0.05, **P < 0.01, ***P < 0.001; ns no significance.

The lipoplex-based systems such as Lipofectamine are useful to easily study RNA interference functionality in vitro, but due to the strong positive charge of the employed lipids, they exhibit cytotoxic effects and are not clinically usable^18^. We therefore generated clinically functional LNP that contain the cationic aminolipid KL-52 using a T-junction. The lipids XL52, 1,2-distearoyl-3-phosphatidylcholine (DSPC), cholesterol, and α-[3′-(1,2-dimyristoyl-3-propanoxy)-carboxamide-propyl]-ω-methoxy-polyoxyethylene (PEG-c-DOMG) were mixed at molar ratios of 50:10:38.5:1.5 and at a total lipid to siRNA ratio of 7:1. This method is based on mixing two streams whereas one stream contains the lipids in ethanol and the other stream the RNA molecules in an aqueous buffer. By self-assembly, the cationic lipids form complexes containing the siRNA molecules (Fig. 3a).

The most potent *siRNA-JNK2* polymer 3 was encapsulated into LNP for in vivo experiments. The LNP were carefully characterized to ensure its stability and encapsulation of siRNA. The size of the generated LNP was determined as 80.3 nm and the polydispersity index (PDI) was calculated as 0.05, as determined using dynamic light scattering (DLS). The LNP exhibited a neutral surface charge of −0.8 mV. The concentration was 0.42 mg/mL and the drug encapsulation efficiency was 88% as measured using an oligo green assay.

The LNP were injected intravenously into the mice and 6 h after administration, tissue specificity was analyzed by determining *Jnk2* mRNA expression first in different organs. It was discovered that the *siRNA-Jnk2* LNP set three downregulated *Jnk2* mRNA significantly in the liver (Fig. 3b). Therefore, organ specificity, which is a major criterion for targeting liver cells, was given. The next aim was to identify the target cell in which the siRNA accumulates. We therefore isolated different primary cells from liver: hepatocytes (HC), Kupffer cells (KCs), and hepatic stellate cells (HSC). We found that the siRNA was specifically active in hepatocytes and significantly reduced *Jnk2* mRNA expression in HC, but not in KC or HSC (Fig. 3c). These results confirmed successful *Jnk2* mRNA inhibition via *siRNA-Jnk2* set 3 formulated as LNP (hereafter referred as *siJnk2-*LNP) in hepatocytes in vitro and in vivo.

Next, we investigated the dose and time-dependent effect of *siRNA-Jnk2* LNP on mRNA and protein expression in WT mice. Using different siRNA concentrations ranging from 0.05 to 0.2 mg/kg mouse body weight (BW), mRNA and protein expression showed a dose-dependent downregulation of *Jnk2* compared to *siLuc*-treated mice, 6 h after injection. A single dose injection of 0.2 mg/kg per mouse BW triggered almost undetectable JNK2 protein expression after 168 h, although mRNA expression levels showed an increase, compared to the earlier timepoint. Based on these data, a single dose injection of 0.2 mg/kg *siJnk2-*LNP was used in all further experiments (Fig. 3d). We next confirmed, at the protein level, inhibition of total JNK2, while some residual activation of JNK (phospho-JNK) was observable in the livers of siRNA-injected mice (Fig. 3e). This effect was due to the protein levels of JNK1 both in hepatocytes and non-parenchymal cells and JNK2 in non-parenchymal cells (e.g., Kupffer cells or HSC). As observed in the JNK immunoblot, we excluded a compensatory overexpression of JNK1 after downregulation of JNK2.

### Hepatocyte-specific siRNA-dependent Jnk2 deletion in an early phase of chronic liver injury

**Fig. 4:**
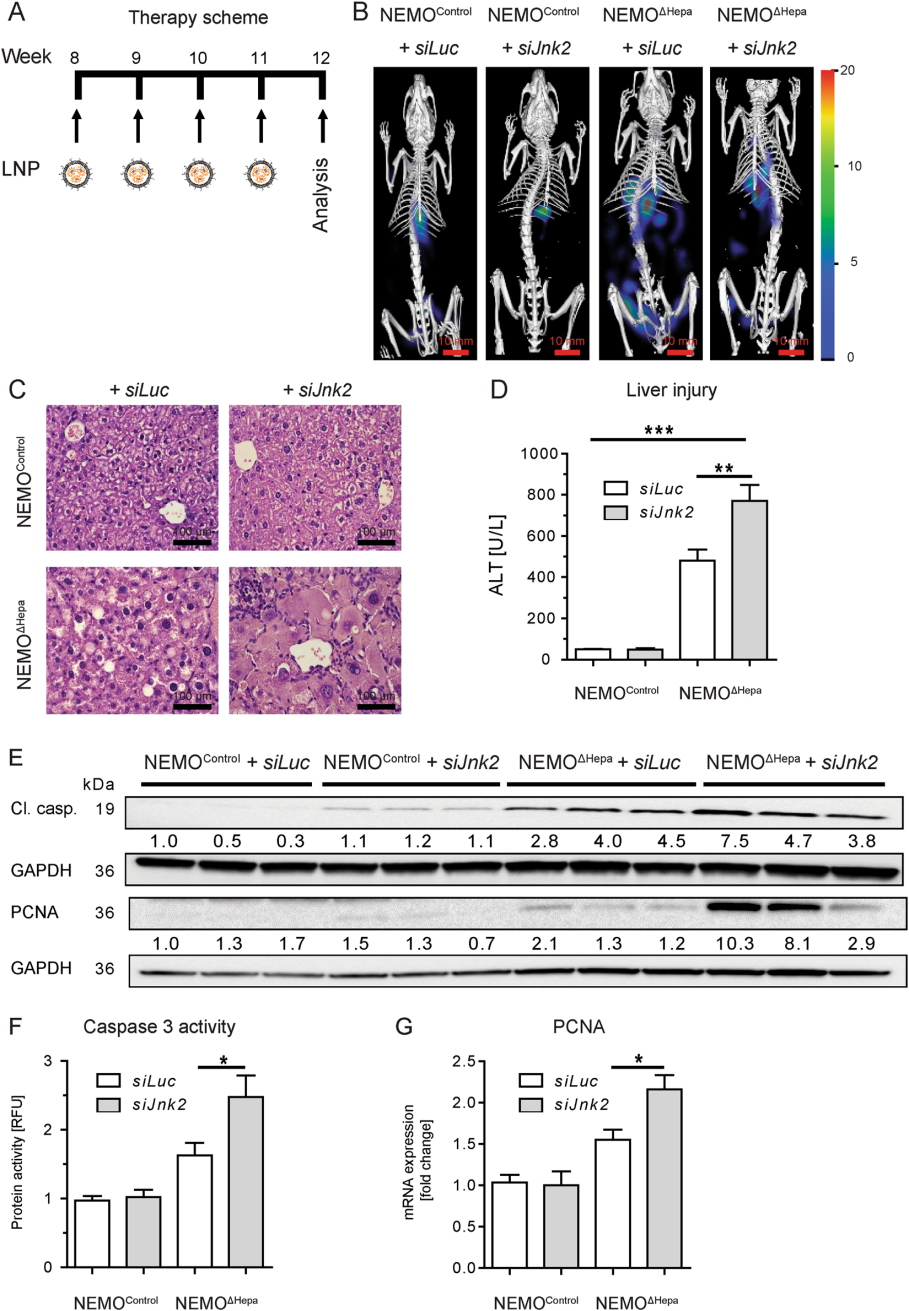
Hepatocyte-specific siRNA-dependent *Jnk2* deletion in an early phase of chronic liver injury. Male 8-week-old NEMO^Control^ and NEMO^ΔHepa^ animals were injected on a weekly basis with a single dose of 0.2 mg/kg *siLuc* or *siJnk2*-LNP, over a period of 4 weeks and sacrificed at 12 weeks of age (*n* = 12). **a** Schematic cartoon of the *siJnk2-*LNP injection protocol. **b** Accumulation of Duramycin-NIR790 in the liver, a quantitative biomarker of cell death. **c** Representative H&E-stained liver sections prepared from 12-week-old NEMO^Control^ (upper panels) and NEMO^ΔHepa^ (lower panels) mice receiving either *siLuc-*LNP or *siJnk2-*LNP injections. (Scale bars: 100 μm). **d** Liver injury reflected by alanine aminotransferase (ALT) enzyme activity. **e** Immunoblot analysis of whole liver extracts detecting cleaved caspase-3 and PCNA. GAPDH was used as loading control. **f** Caspase-3 activity in the livers from 12-week-old NEMO^Control^ mice compared to NEMO^ΔHepa^ mice receiving *siLuc-*LNP or *siJnk2-*LNP injections. The activity was represented as relative fluorescence units (RFU). **g** Hepatic mRNA levels of PCNA were quantified and displayed as fold increase for the identical treatment groups. Data are presented as means ± S.E.M. *P < 0.05, **P < 0.01, ***P < 0.001.

To study the role of *Jnk2* inhibition at different stages of liver disease, we studied NEMO^ΔHepa^ mice at an early stage of liver disease, in which tumor development is not present. We performed four weekly injections into NEMO^ΔHepa^ mice at the dose of 0.2 mg/kg *siJnk2*-LNP or *siLuc-*LNP and studied the effects on CLD progression (Fig. 4a). Apoptotic cell death is mediated by activity of the enzyme caspase-3 and plays a major role in CLD of NEMO^ΔHepa^ mice^19^. To validate the change in caspase-3 activity and thereby apoptotic cell death in *siJnk2-*LNP-treated mice, animals were injected duramycin-NIR790 fluorescent conjugate and subsequently FMT/μCT was performed. The near infrared (NIR) Cy7-type dye is attached to amino groups of the polypeptide antibiotic duramycin. In addition, the conjugate contains one Cy7 dye molecule per duramycin molecule. Upon injection, duramycin binds phosphatidylethanolamine (PE) at a 1:1 ratio with high affinity and exclusive specificity. Under normal cell conditions, PE is restricted to the inner part of the cell membrane. Upon apoptosis, PE is exposed to the outer plasma membrane bilayer and thereby bound by Duramycin. Duramycin-based imaging probes have been successfully applied for the non-invasive imaging of cell death including apoptosis in disease diagnosis and for therapy monitoring^20^. In vivo imaging using FMT/µCT demonstrated that the amount of Duramycin-NIR790 conjugate bound to phosphatidylethanolamine in apoptotic cells was markedly increased supporting the histological analysis (Fig. 4b, Supplementary Fig. 2a).

Histopathological analysis of our experimental model revealed significant differences between *siJnk2-*LNP and *siLuc-*LNP-treated NEMO^ΔHepa^ livers. Silencing of *Jnk2* in hepatocytes of 12-week-old NEMO^ΔHepa^ mice triggered parenchymal cell dysplasia and hypertrophy accompanied with strong anisokaryosis. In addition, there was increased fat deposition and substantial immune cell infiltration. Immune cells were found in local clusters throughout the liver parenchyma (Fig. 4c, Supplementary Figs. 2d, e and 3a). Treatment with *siJnk2-*LNP for 28 days significantly impaired liver injury markers compared to *siLuc-*LNP-treated NEMO^ΔHepa^ mice (Fig. 4d, Supplementary Fig. 2b). Interestingly, *siJnk2-*LNP treatment led to a decrease in liver/body weight (LW/BW) ratio in 12-week-old NEMO^ΔHepa^ animals (Supplementary Fig. 2c).

In the same direction, *siJnk2-*LNP*-*treated NEMO^ΔHepa^ livers showed significantly increased cleaved caspase-3 levels, a marker of apoptosis, compared to *siLuc*-LNP treated animals as evidenced by both western blot analysis (Fig. 4e) and immunohistochemistry (Supplementary Fig. 3b). Quantification of Caspase-3 activity in liver homogenates confirmed increased apoptosis in *siJnk2*-LNP-treated NEMO^ΔHepa^ livers (Fig. 4f). Next, proliferation activity measured by PCNA expression was determined as a sign of liver repair and regeneration. Our data revealed significantly increased parenchymal and non-parenchymal cells stained positive for PCNA at the protein and mRNA levels in NEMO^ΔHepa^ livers after *siJnk2-*LNP treatment (Fig. 4g, Supplementary Fig. 3c). To corroborate these results, we performed Ki67 immunofluorescence, which showed significantly increased Ki67-positive cells in non-parenchymal cells in *siJnk2*-LNP-treated NEMO^ΔHepa^ livers (Supplementary Fig. 3d).

### Treatment of advanced stage liver tumors with lipid nanoparticles

**Fig. 5:**
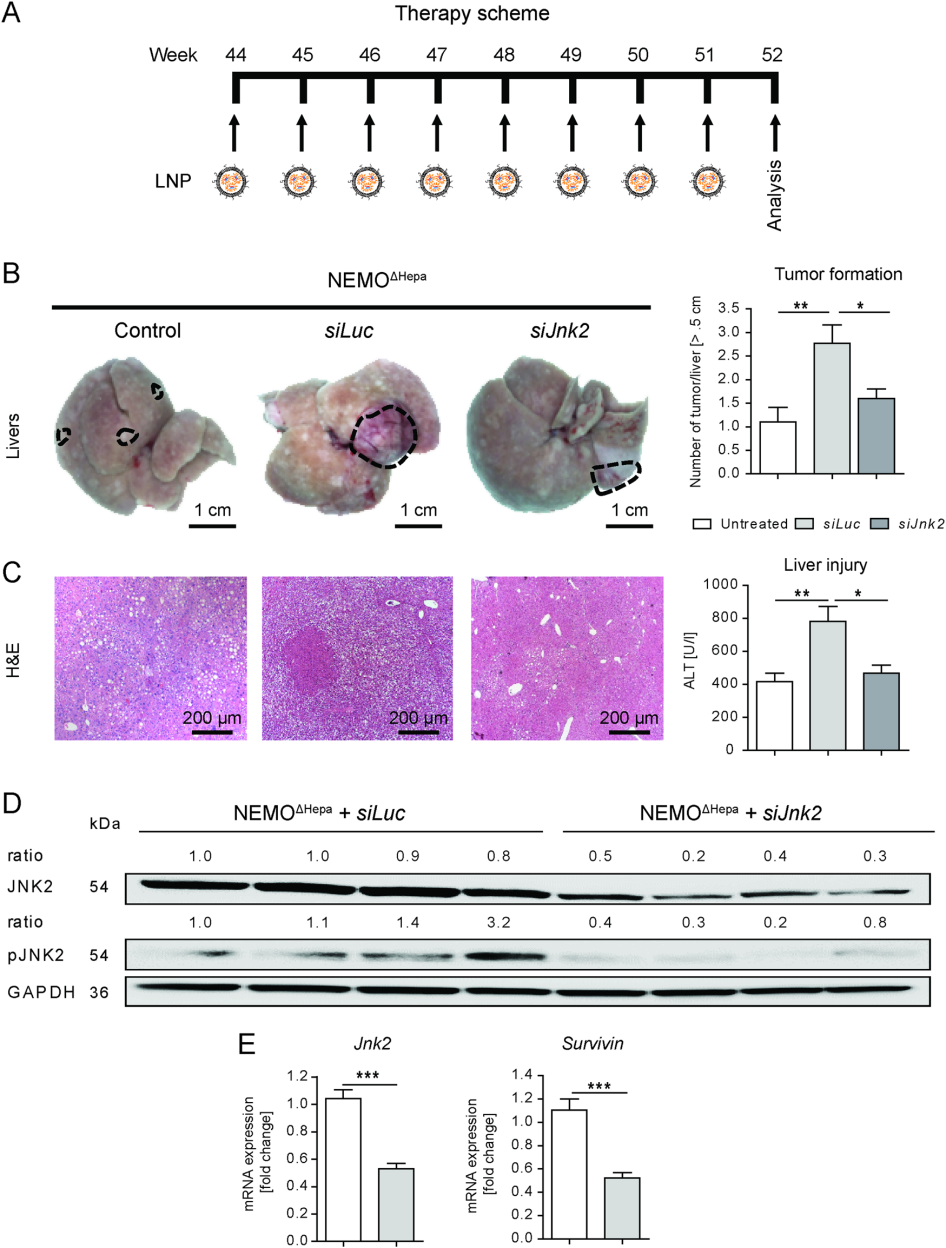
Treatment of advanced stage liver tumors with lipid nanoparticles targeting *Jnk2* in Hepatocytes. Male 44-week-old NEMO^ΔHepa^ mice were injected weekly with a single dose of 0.2 mg/kg luciferase (*siLuc*) or *siJnk2*-LNP beginning at 44 weeks of age until reaching 52 weeks of age. **a** Experimental setup of the weekly injections of the LNP. **b** Representative macroscopic images (left) of the livers of NEMO^ΔHepa^ mice at an age of 44 weeks (control) and with 52 weeks. Dashed circles indicate tumoral lesions (scale bars: 1 cm). Number of tumor nodules ≥0.5 cm in diameter (right) were quantified. **c** Representative H&E staining of liver sections (left) of untreated 44-week-old mice and the two treatment groups after *siRNA-*LNP treatment. Liver injury reflected by serum ALT (right, *n* = 12–14). **d** Expression of JNK2 and pJNK2 was analyzed by immunoblotting of liver samples of one-year-old NEMO^ΔHepa^ mice treated with either *siLuc*-LNP or *siJnk2-*LNP. **e** Quantitative RT-PCR analysis of the mRNA levels of *Jnk2* and *survivin* for the same NEMO^ΔHepa^ treatment groups were determined and are represented as fold increase. Data are presented as means ± S.E.M. *P < 0.05, **P < 0.01, ***P < 0.001.

Cancer progression occurs in different stages and inflammatory processes may have a stage-dependent role^11^. We therefore sought to study the impact of inhibiting *Jnk2* at late stages of liver disease where tumors are still apparent in the NEMO model. Thus, we investigated the effect of hepatocyte-specific *Jnk2* silencing at a later stage of disease progression. Therefore, 44-week-old NEMO^ΔHepa^ mice were treated with *siJnk2-*LNP for 8 weeks and subsequently analyzed (Fig. 5a).

Chronic *siJnk2-*LNP treatment of NEMO^ΔHepa^ mice caused a significant reduction in disease progression. *siJnk2*-LNP treatment led to improved architecture of the hepatic parenchyma associated with minor signs of steatosis, a reduction of well-differentiated HCCs and significantly less premalignant dysplastic nodules, associated with significantly decreased liver serum ALT levels (Fig. 5b, c, Supplementary Fig. 4a).

Overall, gene silencing of *Jnk2* translated into decreased tumor numbers (HCC initiation) and improved liver parenchyma compared to untreated, 44-week-old NEMO^ΔHepa^ mice. Macroscopic analysis and H&E staining of *siLuc*-LNP-treated NEMO^ΔHepa^ liver displayed HCCs exhibiting dysplastic nodules as well as differentiated adenomas with characteristic neovascularization (neoangiogenesis) (Fig. 5b, c, Supplementary Fig. 4b). In-line with improved liver architecture, *siJnk2*-LNP treatment of NEMO^ΔHepa^ livers showed significantly reduced collagen accumulation evidenced by SR analysis (Supplementary Fig. 4c).

Finally, to confirm that downregulation of JNK2 by *siJnk2-*LNP treatment caused the improved tumorigenesis in experimental CLD, we analyzed both JNK2 protein and mRNA expression (Fig. 5d, e). Western Blot analysis revealed a downregulation of JNK2 and pJNK2 protein expression (Fig. 5d). Quantitative realtime-PCR (qPCR) confirmed reduced expression of *Jnk2* mRNA transcripts, and also of *survivin*, a gene highly expressed in tumors (Fig. 5e).

### Effects of Jnk2-directed siRNA lipid nanoparticles on cell death in mice with advanced liver disease and tumorigenesis

**Fig. 6:**
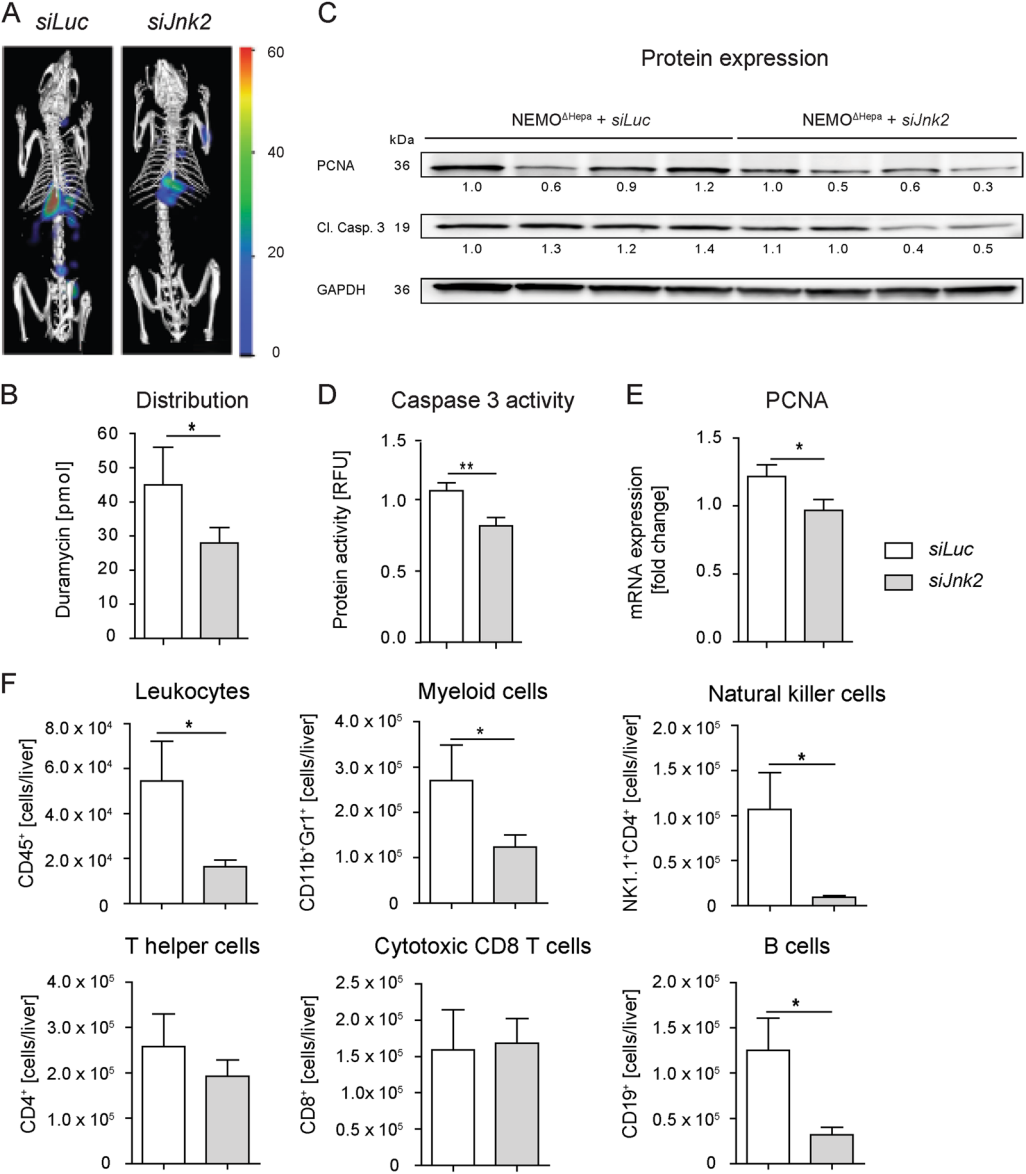
Effects of Jnk2-directed siRNA lipid nanoparticles on cell death in mice with advanced liver cancer. Mice aging 44 weeks were treated with either *siLuc* or *siJnk2*-LNP for another 8 weeks. **a** 52-week-old NEMO^ΔHepa^ mice were injected with Duramycin-NIR790 and non-invasive µ-CT-FMT-based whole body imaging was performed. Representative FMT/µCT images showing the accumulation of Duramycin-NIR790 in the liver. **b** µCT-FMT-based quantification of Duramycin in livers. **c** Liver tissue extracts were subjected to immunoblotting for PCNA and cleaved Caspase-3. The same membrane was then stripped and reblotted for GAPDH, used as loading control. **d** Activity of cleaved caspase 3 and PCNA **e** mRNA expression. **f** Flow-cytometry analysis demonstrated changes in immune cell populations. Cells were counted and quantified and given as cell numbers for the whole liver. Data analysis is expressed as means ± S.E.M. *P < 0.05, **P < 0.01, ***P < 0.001.

To study the role of hepatocytes cell death, triggered by inflammatory processes enhanced by JNK2, we administered duramycin-NIR790 fluorescent conjugate. The µCT-FMT images demonstrated a reduced signal of Duramycin and hence, reduced cellular apoptosis in the NEMO^ΔHepa^ mice treated with *siJnk2-*LNP (Fig. 6a). Quantifications of the µCT-FMT data confirmed reduced apoptotic cell death in NEMO^ΔHepa^ livers after *siJnk2-*LNP injection compared to *siLuc*-LNP-treated animals (Fig. 6b).

In order to study further apoptosis and proliferation in the livers, we performed western-blot-based immunoblotting and studied cleaved caspase-3 and compensatory hepatocyte proliferation by PCNA protein levels (Fig. 6c). Analysis of caspase-3 activity demonstrated, in accordance to the Duramycin imaging, reduced apoptotic cell death by *siJnk2*-LNP treatment (Fig. 6d). A significant reduction in PCNA mRNA levels demonstrated reduced proliferation of hepatocytes (Fig. 6e).

We further performed multicolor flow cytometry for immune cells of these livers. NEMO^ΔHepa^ mice inoculated with *siJnk2-*LNP livers showed significantly reduced number of infiltrating CD45^+^ cells. Using a bead-based quantification of the cells, we determined the cell number per liver, which reflects the total amount of the cells in the liver. We found that inhibition of *Jnk2*, but not of *Luc*, significantly reduced total numbers of CD45^+^ cells as well as myeloid cells, natural killer (NK) cells, and B cells. The antitumoral T cells, both CD4 and particularly the cytotoxic CD8 T cells, which can kill tumor cells, were unaffected by the *siJnk2-*LNP (Fig. 6f, Supplementary Fig. 4d).

## Discussion

Nanomedicines open novel therapeutic options for diseases with huge unmet medical need. Novel drug formulations offer therapeutic approaches for treatment of liver diseases, including inflammation, fibrosis, or cancer^21–23^. However, antibody-mediated tumor cell targeting has been shown to be accompanied with difficulties^24^. The combination of RNA delivery particularly offers remarkable potential; however, it has faced difficulties in the past^17^. Using cationic lipids enable an efficient delivery and have been demonstrated to target liver cells in vivo^25^. In liver disease, different etiologies e.g., viral hepatitis, alcohol, or metabolic liver diseases are known to trigger CLD and ultimately end-stage liver disease, including occurrence of HCC^26^. The incidence of liver cancer as a cause of death is globally increasing. However, limited treatment options are currently available and hence there is a rising need to understand molecular pathways involved in CLD and liver cancer pathophysiology.

Previous studies from our group demonstrated that the NF-κB pathway and activation of JNKs play a major role in HCC development^12,13^. Therefore, the aim of this study was to investigate the outcome of downregulating *Jnk2* exclusively in hepatocytes in an experimental model of CLD, the NEMO^ΔHepa^ mice. Moreover, we used two different experimental approaches: a genetic knockout and an inducible knockdown using siRNA technology, respectively. These approaches are different but complementary. A complete loss of protein function was achieved using double-knockout mice. However, this is a time and costly experimental approach and gene deletion during embryonal development may cause non-physiological compensatory pathways. In contrast, the knockdown approach using siRNA allows a quicker on demand evaluation of mechanisms governed by *Jnk2*, reflecting more closely the human situation, since patient’s liver diseases are the result of changes in gene and/or protein expression.

First, the analysis of genetic double-knockout mice for JNK2^ΔHepa^ and NEMO^ΔHepa^ (NEMO/JNK2^ΔHepa^) concurred with our previous findings that *Jnk2* knockout in NEMO^ΔHepa^ mice showed an improved outcome in HCC development^13^. Consistent with decreased liver transaminases, NEMO/JNK2^ΔHepa^ mice livers showed reduced overall number of tumors. These results suggest that hepatocyte-specific deletion of JNK2 improves tumor progression but impairs tumor initiation and thus we next questioned whether JNK2 inhibition could be a therapeutic approach for the development of CLD. Concomitant with earlier observations^16^, the efficacy of LNP targeting specifically hepatocytes in vivo was demonstrated. Specifically, we established a genetic knockdown for *Jnk2* exclusively in hepatocytes by taking advantage of the characteristics of LNP and its uptake by hepatocytes via LDLRs. Therefore, we focused on CLD at different stages of disease progression in our experimental model.

Interestingly, *siJnk2-*LNP treatment in NEMO^ΔHepa^ mice during the late phase of CLD disease revealed significant overall improvement. Reduced liver serum values and significantly improved serum biochemical parameters were supported by liver histopathology studies. At late stages of CLD, *Jnk2* downregulation exerted a protective role as demonstrated by ameliorated progression of CLD shown by reduced fibrogenesis. The reduced fibrogenic response directly translated in decreased tumor initiation suggesting that the carcinogenic environment after *siJnk2*-LNP treatment improved in NEMO^ΔHepa^ livers as also reflected by improved liver architecture. Most importantly, *siJnk2-*LNP treatment prevented HCC progression, characteristic of NEMO^ΔHepa^ livers at an age of 44 weeks.

Interestingly, this observation was associated with a strong impact on the adaptive immune system, showing changes in T helper and NKT cells. CD4^+^ and CD8^+^ T-cells have opposing roles in promoting a chronic proinflammatory environment and triggering antitumor surveillance. However, their role in HCC initiation is still a matter of debate and confounding data using different experimental models has been reported. Interestingly and in agreement with improved disease progression after *siJnk2*-LNP treatment, FACS analysis underscored an antitumor effect for both subpopulations on HCC.

Nevertheless, our data suggest that stage-dependent interventions and personalized analyses of liver tumors may enhance therapeutic effects. Interestingly, *Jnk2* downregulation in 12-week-old NEMO^ΔHepa^ mice caused a proinflammatory environment aggravating the phenotype of NEMO^ΔHepa^ mice at this timepoint. *Jnk2* siRNA-mediated nanodelivery caused exacerbated hepatic damage, immune cell infiltration, impaired liver fibrosis, apoptosis, and compensatory proliferation.

Analyzing immune cell infiltration revealed cells as monocyte-derived macrophages (MoMFs) by different methods confirm previous observations that hepatic macrophage numbers are strongly increased independent of the type of liver injury. Remarkably, not only hepatocytes but also infiltrating cells were positively stained for cleaved Caspase-3 and undergoing apoptosis after *siJnk2*-LNP treatment. Kupffer cells (KCs) have been described as long-lived resident macrophages, even though a constant turnover occurs and hepatic macrophages are incessantly repopulated^27^. Since the intrahepatic macrophage number is massively expanded following the influx of peripheral monocytes rather than augmentation of tissue-resident macrophages, the observed results offer indisputable evidence for a high turnover and consistently repopulation of MoMFs due to hepatocyte-specific *siJnk2*-LNP treatment in NEMO^ΔHepa^ mice.

Our findings provide clear evidence that lipid-based RNAi drugs are ideally suited for cell therapy of end-stage liver cancer. Usage of a selective cationic lipid has enabled to efficiently transfer the siRNA into hepatocytes, thus impeding the initiation of liver cancer. Comparable RNA delivery systems are succeeding in the clinics and it is very likely that a larger number of RNA-based medicines will help to cure diseases where treatment options are still rare, including hepatic carcinogenesis.

Most importantly, we present a novel treatment strategy for advanced liver cancer, which is frequently diagnosed. At this stage of the cancer, therapeutic options are very limited and growth arrest or size reduction of tumors is highly desirable. Based on our murine data, our study highlights the success of siRNA-based therapy during CLD. The NEMO mouse is a suitable experimental model to study the progression of liver disease, starting with cell death, compensatory proliferation, NASH, and end-stage HCC^12^. Our results indicate that *siJnk2* therapy would be most successful when administered in advanced stages of liver disease once steatohepatitis is detected, suggesting that patients with advanced liver fibrosis and/or NASH could benefit from siRNA therapy in the clinic.

Overall, our findings define a pivotal time-dependent role of *Jnk2* during the development of experimental CLD. In particular, hepatocytic siRNA-mediated *Jnk2* inhibition in mice with advanced cancer-blocked HCC progression. Hence, these results define *Jnk2* as a potential preventive approach targeting hepatocytes to impair cancer initiation in chronically damaged livers. Moreover, similar phenotypes observed in both the knockout and the knockdown mice highlight the usefulness of the siRNA technology. Most importantly, the formulated lipid nanoparticles are organ-specific by size and cell-type-specific due to intrinsic effects of the cationic aminolipid.

## Methods

### Materials

The lipid 1,2-distearoyl-3-phosphatidylcholine (DSPC) was purchased from Avanti Polar Lipids (Alabaster, Albama, USA) and the PEG lipid α-[3′-(1,2-dimyristoyl-3-propanoxy)-carboxamide-propyl]-ω-methoxy-polyoxyethylene (PEG-c-DOMG) was obtained from NOF (Bouwelven, Belgium). Cholesterol was purchased from Sigma–Aldrich (Taufkirchen, Germany). The siRNA polymers were designed based on their ability to specifically target *Jnk2* in mice, specifically in hepatocytes. In addition, mismatches to *Jnk1* (2–18 nucleotides) were incorporated, to increase in vivo stability and suppression of the immune-stimulatory properties, resulting in 12 different siRNA sets. The siRNA was purchased from Axolabs GmbH (Kulmbach, Germany).

### Generation of lipid-based nanoparticles

Stock solutions of KL-52 aminolipid, DSPC, cholesterol, and PEG-c-DOMG were prepared at concentrations of 50 mM in ethanol and stored at −20 °C until use. The lipids were mixed in ethanol at molar ratios of 50:10:38.5:1.5 (KL-52: DSPC: Cholesterol: PEG-c-DOMG) and diluted with ethanol to a final lipid concentration of 25 mM. The siRNA stock solutions were prepared at a concentration of 10 mg/mL in H_2_O and were diluted in 50 mM sodium citrate buffer with pH 3. The lipid nanoparticles (LNP) were prepared by mixing the lipid with the siRNA solution at a total lipid to siRNA weight ratio of 7:1 using a T-junction mixer. The lipid ethanolic solution was rapidly injected into the aqueous siRNA solution, leading to a suspension containing 33% ethanol. The solutions were pumped by using a syringe pump (Harvard Pump 33 Dual Syringe Pump Harvard Apparatus Holliston, MA).

Subsequently, the formulations were dialyzed against phosphate buffered saline (PBS), pH 7.4 at volumes 200-times of the primary product using Slide-A-Lyzer cassettes (Thermo Fisher Scientific Inc. Rockford, IL) with a molecular weight cutoff (MWCO) of 10 kD to remove free siRNA and ethanol. The first dialysis step was done at room temperature for 3 h and then the formulations were dialyzed overnight at 4 °C. The resulting nanoparticle suspension was filtered through a 0.2 µm sterile filter (Sarstedt, Nümbrecht, Germany) into glass vials and sealed with a crimp closure.

### Characterization of lipid-based nanoparticles

Particle size and zeta potential of formulations were determined using a Zetasizer Nano ZS (Malvern Instruments Ltd, Malvern, Worcestershire, UK) in 1X PBS and 15 mM PBS, respectively. The siRNA concentration in the liposomal formulation was measured by UV-vis spectrophotometry. Encapsulation of siRNA by the nanoparticles was evaluated by the Quant-iT™ RiboGreen^®^ RNA assay (Invitrogen Corporation Carlsbad, CA).

### Knockout mouse strains and animal housing

Male mice carrying the loxP-site-flanked under the control of the Alb/AFP-Cre promotor/enhancer were generated as previously described^28^. Mice lacking the Cre promoter were named as “Control” mice (e.g., NEMO^Control^), showing no functional difference to WT mice. Albumin-Cre (Alb-Cre), *Jnk2*-deficient mice were purchased from The Jackson Laboratory (Bar Harbor, ME, USA). Hepatocyte-specific *Jnk2* and NEMO/IKKγ mice were constructed by crossing Alb-Cre JNK2^Control^ mice with Alb-Cre NEMO^Control^ mice. All strains were crossed on a C57BL/6 background. The mice were housed in the Institute of Laboratory Animal Science at the University Hospital of RWTH-Aachen University, according to German legal requirements (Deutsches Tierschutzgesetz, FELASA, GV-SOLAS) under a permit of the ‘Veterinäramt der Städteregion Aachen’. In addition, the mice were kept in individual ventilated cages (IVC) in groups of maximum five animals per cage under specific pathogen free (SPF) conditions. The mice were examined on a daily basis by animal caretakers of the Institute of Laboratory Animal Science at the University Hospital, RWTH-Aachen University. Specific air conditioning assured a constant room temperature of about 22 ± 1 °C and humidity in the range of 50 ± 10%. At a light source of 150 lux, the mice were housed in a 12 h light–dark cycle (7:00–19:00 light; 19:00–7:00 dark). Mice received autoclaved food pellets and sterilized water both ad libitum. For breeding, the mice were mated at around 9 weeks and the littermates were separated at the age of 3–4 weeks following earmarks for identification. All organ explants and animal experiments were approved by the local authority for environment conservation and consumer protection of the state North Rhine Westphalia (LANUV) on the following animal grants: 30034 G (AZ: 84–02.04.2016.A080), TVA-11324GZ (AZ-84-02.04.2016.A490). Furthermore, the research was performed under the ARRIVE (Animal Research: Reporting of In Vivo Experiments) guidelines.

### Non-invasive experimental imaging

The mice were shaved 1 day before the experiment at the abdomen and back with an electric shaver to reduce any possible interference by hairs during the FMT scans. In the next step, 9 h before the FMT/µCT scan the mouse was injected intravenously with the cell death probe Duramycin-NIR790 conjugate (66.7 pmol/kg, kindly provided by Chris Pak, Molecular Targeting Technologies, Inc.).

### Isolation and culture of liver cells

Hepa 1–6 (1 × 10^5^ cells/well) cells were obtained from the American Tissue Culture Collection (ATCC, Manassas, VA, USA), grown in DMEM (Gibco BRL, Grand Island, NY, USA) supplemented with 10% fetal bovine serum (FBS) and 1% penicillin/streptomycin. Hepa1–6 cells were cultured in a quantity of 500.000 cells/well, on 6 well 15.5 ml/9.6 cm^2^ culture plates (Falcon, Corning Inc., Corning, NY, USA). Primary murine hepatocytes from C57BL6 mice were isolated from 7–8-week-old mice by collagenase perfusion. Living hepatocytes were plated on collagen-precoated petri dishes at a density of 1.5 × 10^4^/cm^2^ in supplemented DMEM medium and after 4 h incubation (37 °C, 5% CO_2_) medium was renewed. Hepatic stellate cells (HSCs) and Kupffer cells (KCs) were isolated according to the published protocol^29^.

### Cell transfection in vitro

Cells were transfected with the corresponding siRNA molecules according to the manufacturer’s protocol (Lipofectamine^®^ RNAiMAX™ Transfection Reagent, ThermoFisher Scientific) and cultured for 24 h at 37 °C. After this, RNA was isolated (PureLink RNA Mini Kit, ThermoFisher Scientific) and analysed.

### Statistical analysis

All data were analyzed by Graph Pad Prism^®^ 5.0 (GraphPad Software, Inc.) and are expressed as means ± standard error of the mean (SEM). *P*-values below 0.05 were considered significant using the Student’s *t*-test or by one-way analysis of variance (ANOVA) including Bonferroni post-hoc test.

## Supplementary information

Suppl. Material

Suppl. Fig. 1

Suppl. Fig. 2

Suppl. Fig. 3

Suppl. Fig. 4
