# Supplementary material for: Lipid-encapsulated siRNA for hepatocyte-directed treatment of advanced liver disease
Source: Cell Death Dis. 2020 May 11;11(5):343. doi: 10.1038/s41419-020-2571-4 (PMC7214425; doi:10.1038/s41419-020-2571-4)
Supplement: Supplementary file 1 — Suppl. Material [file 41419_2020_2571_MOESM1_ESM.docx]

SUPPLEMENTARY DATA

**CDDIS-19-4234RRR**

Lipid-encapsulated siRNA for hepatocyte-directed

treatment of advanced liver disease

Marius Maximilian Woitok^1*^, Miguel Eugenio Zoubek^1,3*^, Dennis Doleschel^2^, Matthias Bartneck^1^, Mohamed Ramadan Mohamed^1^, Fabian Kießling^2^, Wiltrud Lederle^2^, Christian Trautwein^1#^, Francisco Javier Cubero^1,4,5#^

# **SUPPLEMENTARY MATERIAL AND METHODS**

**Immunohistochemistry staining.** Liver tissue slides were stained for Cleaved Caspase 3 (Cell Signaling Technology), PCNA (Dianova GmbH) on paraffin sections. Eight randomly chosen images were taken of each liver tissue section (20X magnification, scale bars: 100 µm) for analysing using Axiovision software (Carl Zeiss, Jena, Germany).

**Immunofluorescence staining Ki-67.** Liver tissue cryosections were fixed in 4% PFA for 10 min. Samples were rinsed in PBS-Tween for 10 min after fixation and blocked with 10% goat serum for 1h. Subsequently, incubation with primary antibody Ki-67 (Leica Biosystems Inc., Leider Lane, IL, USA) was performed at 4°C overnight. The day after, samples were incubated with appropriate fluorescence-labelled secondary antibodies (AlexaFluor 488 and 564, Invitrogen, Oregon, USA) for 1 h at room temperature. After the incubation time, the sections were washed by three repetitive 3 min washing steps in PBS-Tween and were counterstained with mounting medium with DAPI. Subsequently, the sections were assed under the microscope.

**Immunofluorescence staining CD11b+F4/80+.** First, freshly cut sections were fixed with formaldehyde (4%) for 10 min followed by 3x 3 min washing with DPBS+0,02% NaAzide. Next, the sections were blocked with DPBS+ 0,02% NaAzide+ 0,2% BSA for 5 min at room temperature. The blocking solution was removed by 3x 5 min washing step in DPBS+ 0,02% NaAzide. In the next step, the sections were incubated at room temperature with the primary antibody CD11b (BD, 1:500 in DPBS+ 1% mouse serum) for 45 minutes. Subsequently, the sections were washed 3 x 5 min with DPBS+ 0,02% NaAzide followed by the incubation of the secondary goat anti-rat / Cy3 antibody (1:500 in DPBS+ 1% mouse serum) at room temperature for 1 hour. After the incubation time, the sections were washed by three repetitive 3 min washing steps in DPBS+ 0,02% NaAzide.

To obtain a double staining, the sections were blocked again with DPBS+ 0,02% NaAzide+ 0,2% BSA for 5 min at room temperature. In the next step, the sections were incubated over night at 4°C with the primary F4/80 antibody (Bio-Rad, 1:200 in DPBS+ 1% mouse serum). The next day, the sections were washed for 3 x 5 min with DPBS+ 0.02% NaAzide followed by the incubation of the secondary goat anti-rat / Alexa 488 antibody (1:500 in DPBS+ 1% mouse serum) at room temperature for 1 hour. After the incubation time, the sections were washed by three repetitive 3 min washing steps in DPBS+ 0,02% NaAzide and were counterstained with mounting medium with DAPI. Subsequently, the sections were assed under the microscope. Eight randomly chosen images were taken of each liver tissue section (20X magnification, scale bars: 100 µm) for the analysis using Axiovision software (Carl Zeiss, Jena, Germany).

**Fluorescent activated cell sorting (FACS).** To assess the different immune cell type in the liver, the mice were scarified and the approximately 200 mg of the liver was used for preparation to perform FACS analysis.

The liver specimen was digested in 5 ml RPMI medium containing 2 mg Collagenase 2 (Roche) and was incubated for 45 min at 37°C on a shaker. Next, the digested liver was meshed trough a 70 µm cell strainer in a new tube and gently mixed with 10 ml Hanks complete solution (500ml HBSS+ 3 ml 10% BSA+ 1.5 ml EDTA pH 8, 0.5 M). To separate the leukocytes from hepatocytes and cell debris, the sample was centrifuged for 5 min at 50x g, 4°C. Subsequently, the supernatant was transferred into a new tube and the sample was centrifuged for 10 min at 450x g, 4°C to pellet the leukocytes. The supernatant was removed and the pellet was gently mixed with 5 ml lysis buffer (BD Pharm Lyse) for 15 min at 4°C to degrade the remaining erythrocytes in the sample. After this incubation time, the sample was gently mixed with 10 ml Hanks complete solution and centrifuged for 10 min at 450x g, 4°C. Next, the supernatant was removed and the sample was blocked with 200 µl of the FACS blocking solution (10 ml HBSS+ 5 ml 10% FBS) for 20 min at 4°C. After the blocking step, the sample was centrifuged for 10 min at 450x g, 4°C to remove the supernatant containing the FACS blocking solution. Immune cells were stained for the myeloid and lymphoid panel (1/80 dilution) for 30 min at 4°C with the following antibodies: CD11b / PE, CD11c / APC, CD3e / APC, CD4 / PE, CD45 / APC-eFluor® 780, CD8 / FITC, F4/80 / PE-Cy7, NK1.1 / PE-Cy7 (eBioscience), CD19 / Percp-Cy5.5, Gr1-Percp / Cy5.5, Ly6G / FITC (BD).

In the next step, the cell and antibody suspension was centrifuged for 10 min at 450x g, 4°C and resuspended in 200 µl Hanks complete solution. In the last step, each sample was mixed with 20 µl Hoechst (Invitrogen) and 20 µl of approximately 20000 APC-Calibrate-Beads. The samples were placed on ice and were analyzed in the FACS machine (FACS Canto, BD).

**Real-Time quantitative PCR.** Using the Omniscipt® RT Kit (ThermoFisher Scientific), cDNA was analyzed for the following genes. All primers were purchased from Eurofins MWG Operon (Huntsville, USA). The following primer sequences were used for mice genotyping and Data were analyzed using QuantStudio™ (Thermo Scientific) software.

**Combined fluorescence molecular tomography and microcomputed tomography (FMT/μCT).** The fluorescence molecular tomography (FMT) and micro computed tomography (μCT) measurements were performed at the Institute for Experimental Molecular Imaging (ExMI) at the University Hospital Aachen.

First, the mouse was shaved 1 day before the experiment at the abdomen and back with an electric shaver to reduce any possible interference by hairs during the FMT scans. In the next step, 9 hours before the FMT/µCT scan the mouse was injected intravenously with the cell death probe Duramycin-NIR790 conjugate (66.7 pmol/kg, kindly provided by Chris Pak, Molecular Targeting Technologies, Inc.). Duramycin-NIR790 conjugate binds to phosphatidylethanolamine (PE) with high affinity and specificity. Under normal conditions, PE is restricted to the inner part of the cell membrane. Upon apoptosis and cell death, PE is exposed to the outer leaflet of the cell membrane, thereby enabling the binding of the imaging probe. Duramycin-based imaging probes have been successfully applied for the non-invasive imaging of cell death including apoptosis in disease diagnosis and for therapy monitoring.^1^

For scanning, the mouse was anesthetized using isoflurane (2% v/v). To precisely localize the liver and other organs, a µCT scan was performed directly before the FMT measurement (TomoScope 30s Duo, CT Imaging GmbH). During both scans, the mouse was kept anesthetized and was held in a fixed position in a multimodal animal bed. CT imaging was performed using the SQD-6565-360-29 protocol which acquires 720 projections with 516 x 506 pixels requiring a scanning time of 29 s per subscan. Directly after acquiring the µCT scans, the mouse bed was transferred to the FMT system (FMT 2500LX, PerkinElmer), and the FMT scan was performed at 790 nm. Data fusion and reconstruction of the fluorescence distribution were performed as described.^2^ The organs were manually segmented based on the µCT data and the probe concentration in the liver was determined using Imalytics preclinical 2.0 (Gremse-IT GmbH Aachen).^3^

**Microscopic imaging data.** For bright field image acquisition the microscope Imager.A2 with the following objective lenses by Carl Zeiss AG (Oberkochen) were used: Plan-APOCHROMAT 5x/0,16; 10X/0,45; 20x/0,6; 40x/0,95. The camera ‘Axiocam 506 color’ was used for image acquisition. For fluorescent image acquisition the microscope Imager.Z1 with the following objective lense by Carl Zeiss AG (Oberkochen) was used: EC Plan-NEOFLUAR 20x/0,5. The camera ‘Axiocam MRm’ was used for image acquisition. Images were taken at room temperature and the software AxioVision by Carl Zeiss AG (Oberkochen) was used.

**SUPPLEMENTARY TABLES**

# **Supplementary Table 1.** siRNA sequences targeting *Jnk2.* Bioinformatics-based sequence design for the inhibiting *Jnk2* mRNA resulted in 12 different siRNA sets binding to different sequences within the mRNA of JNK2 including possible cross-species reactivity (upper table). From these sequences, 12 different siRNA sets were synthesized and tested first in *in vitro* experiments. A, G, U, C: RNA Nucleotide; a, g, u, c: 2‘-O-methyl-Nucleotide; s: Phosphorothioate; T: desoxy-T residue. siRNA set number 3 was chosen for KL52 lipid nanoparticles (LNP) formulation according to the manufacturer's protocol. For the long-term experiments, a total dose of 0.2 mg/kg BW of *siJnk2*-LNP was dissolved in PBS (PAN-Biotech) and injected *i.v.* (tail vein) once per week. In parallel, a control siRNA against luciferase (siLuc) was used.

|  | **Synthesized siRNA strand** | |
| --- | --- | --- |
| **siRNA set#** | **sense strand sequence (5´-3´)** | **antisense strand sequence  (5´-3´)** |
| 1 | cuuAAAGuGuGucaAucAudTsdT | AUGAUUGAcAcACUUuAAGdTsdT |
| 2 | uaAcuuAuGucAGguuAuudTsdT | AAuAACCUGAcAuAAGUuAdTsdT |
| 3 | cuAGcAAcAuuGuaGuAAAdTsdT | UUuACuAcAAUGUUGCuAGdTsdT |
| 4 | ggAAAGAGcuAAuuuAcAAdTsdT | UUGuAAAUuAGCUCUUUCCdTsdT |
| 5 | gaAAGAGcuAAuuuAcAAAdTsdT | UUUGuAAAUuAGCUCUUUCdTsdT |
| 6 | aaGAGcuAAuuuAcAAAGAdTsdT | UCUUUGuAAAUuAGCUCUUdTsdT |
| 7 | agAGcuAAuuuAcaAAGAAdTsdT | UUCUUUGuAAAUuAGCUCUdTsdT |
| 8 | caccuGAAAuuGAuAcuuudTsdT | AAAGuAUcAAUUUcAGGUGdTsdT |
| 9 | cacuAGGuuAGuuuuuGuudTsdT | AAcAAAAACuAACCuAGUGdTsdT |
| 10 | ggcuuAAuuuucAgccAAAdTsdT | UUUGGCUGAAAAUuAAGCCdTsdT |
| 11 | cauuGGGccuGcAgAcAAAdTsdT | UUUGUCUGcAGGCCcAAUGdTsdT |
| 12 | guGucGuAAuuucaGAcAudTsdT | AUGUCUGAAAUuACGAcACdTsdT |
|  | | |
| *siLuc* | cuuAcGcuGAGuAcuucGAdTsdT | UCGAAGuACUcAGCGuAAGdTsdT |

A, G, U, C: RNA Nucleotide

a, g, u, c: 2‘-O-methyl-Nucleotide

# **SUPPLEMENTARY FIGURES**

# **Suppl. Figure 1.** Serum liver AST (A), GLDH (B) and AP (C) of 52-week-old NEMO^ΔHepa^, JNK2^ΔHepa^**,** NEMO^ΔHepa^/JNK2^ΔHepa^ animals and the respective controls are displayed in U/l (n=11-13). Data are presented as means ± S.E.M. **P*<0.05, ***P*<0.01, ****P*<0.001. (D) Representative images (left) of Sirius red staining of paraffin-embedded 52-week-old NEMO^ΔHepa^, JNK2^ΔHepa^**,** NEMO^ΔHepa^/JNK2^ΔHepa^ (lower images) and control mice (upper images). Quantification of positive Sirius red area fraction (right) was performed with Image J^©^ (Scale bars: 100 μm).

# **Suppl. Figure 2.** (A) The accumulation of Duramycin-NIR790 was determined and expressed as [pmol]. (B) Serum liver AST (left), GLDH (center) and AP (right) of 12-week-old NEMO^ΔHepa^ animals and the respective controls are represented in U/l. (C) Liver weight (left), body weight (center) and liver weight versus body weight ratio (right) is graphed for the identical treatment groups. (D) Representative histological sections of livers from 12-week-old NEMO^ΔHepa^ (lower images) and control mice (upper images) following the administration of either *siLuc* (left) or *siJnk2*-LNP (right) stained with hematoxylin and eosin are shown (Scale bars: 100 μm). (D) Illustrative images of CD11b^+^F4/80^+^stainings by immunofluorescence performed on liver cryoslides of 12-week-old NEMO^control^ (upper images) and NEMO^ΔHepa^ mice (lower images) after being subjected to *siLuc* (left) or *siJnk2*-LNP injections (right). Positive CD11b^+^F4/80^+^ cells are stained green; total cells were counter-stained with DAPI (blue). (Scale bars: 100 μm). Arrows indicate positive cells. Data analysis is expressed as means ± S.E.M. ******P*<0.05, ***P*<0.01, ****P*<0.001.

# **Suppl. Figure 3.** (A) The presence of CD11b^+^/F4/80 cells in the liver were analyzed per IF and quantified as positive cells per view field (left). Absolute number of proinflammatory monocytes (CD45^+^/CD11b^+^ /Gr1.1^+^/F4/80^+^) (center) as well as pro-inflammatory monocyte fraction among CD45^+^ cells (right) following flow cytometry analysis are analyzed. Quantification was determined using FlowJo 7.6. Data are presented as means ± S.E.M. **P*<0.05, ***P*<0.01, ****P*<0.001. Representative images of Cleaved Caspase-3 (B) and PCNA (C) expression on paraffin-embedded liver samples of NEMO^control^ (upper images) and NEMO^ΔHepa^ (lower images) was analyzed by immunohistochemistry at the age of 12-weeks after *siLuc* (left) or *siJnk2*-LNP (right) treatment. Arrows indicate positive cells. (Scale bar: 100 µm). (D) Representative images of Ki-67 stainings by immunofluorescence performed on liver cryoslides of 12-week-old NEMO^control^ (upper images) and NEMO^ΔHepa^ mice (lower images) after treatment with *siLuc* (left) or *siJnk2*-LNP injections (right) and quantification of positive Ki67-positive cells in hepatocytes versus NPC (non-parenchymal cells) (Scale bars: 100 μm). Data are presented as means ± S.E.M. *P<0.05, **P<0.01, ***P<0.001.

**Suppl. Figure 4.** (A) Serum liver AST (left), GLDH (center) and AP (right) of 12-week-old NEMO^Δhepa^ animals and the respective controls are represented in U/l. *(B)* Liver weight (left), body weight (center) and liver weight *versus* body weight ratio (right) is graphed for the identical treatment groups. (C) Representative images of Sirius red staining of paraffin-embedded NEMO^Δhepa^ + *siLuc* and NEMO^Δhepa^ + *siJnk2*-LNP treated mice at 52 weeks of age is shown (left). Quantification of positive Sirius red (SR) area fraction (right) was performed with Image J© (Scale bars: 100 μm). (D) Flow cytometry analysis was performed in whole livers of NEMO^Δhepa^ mice after therapeutic administration of either *siLuc* or *siJnk2*-LNP at the age of 52 weeks. Absolute number of pro-inflammatory cells (CD45+/CD11b+ /Gr1.1+/F4/80+) (left) as well as proinflammatory cell fraction among CD45+ cells (right) were quantified by using FlowJo 7.6. Data analysis is expressed as means ± S.E.M. *P<0.05, **P<0.01, ***P<0.001.

# **SUPPLEMENTARY REFERENCES**

1. Elvas F, Stroobants S, Wyffels L. Phosphatidylethanolamine targeting for cell death imaging in early treatment response evaluation and disease diagnosis. *Apoptosis* 2017, **22**(8)**:** 971-987.

2. Gremse F, Theek B, Kunjachan S, Lederle W, Pardo A, Barth S*, et al.* Absorption reconstruction improves biodistribution assessment of fluorescent nanoprobes using hybrid fluorescence-mediated tomography. *Theranostics* 2014, **4**(10)**:** 960-971.

3. Gremse F, Stärk M, Ehling J, Menzel JR, Lammers T, Kiessling F. Imalytics Preclinical: Interactive Analysis of Biomedical Volume Data. *Theranostics* 2016, **6**(3)**:** 328-341.
