# Supplementary figures and images for: Lipid-encapsulated siRNA for hepatocyte-directed treatment of advanced liver disease
Source: Cell Death Dis. 2020 May 11;11(5):343. doi: 10.1038/s41419-020-2571-4 (PMC7214425; doi:10.1038/s41419-020-2571-4)

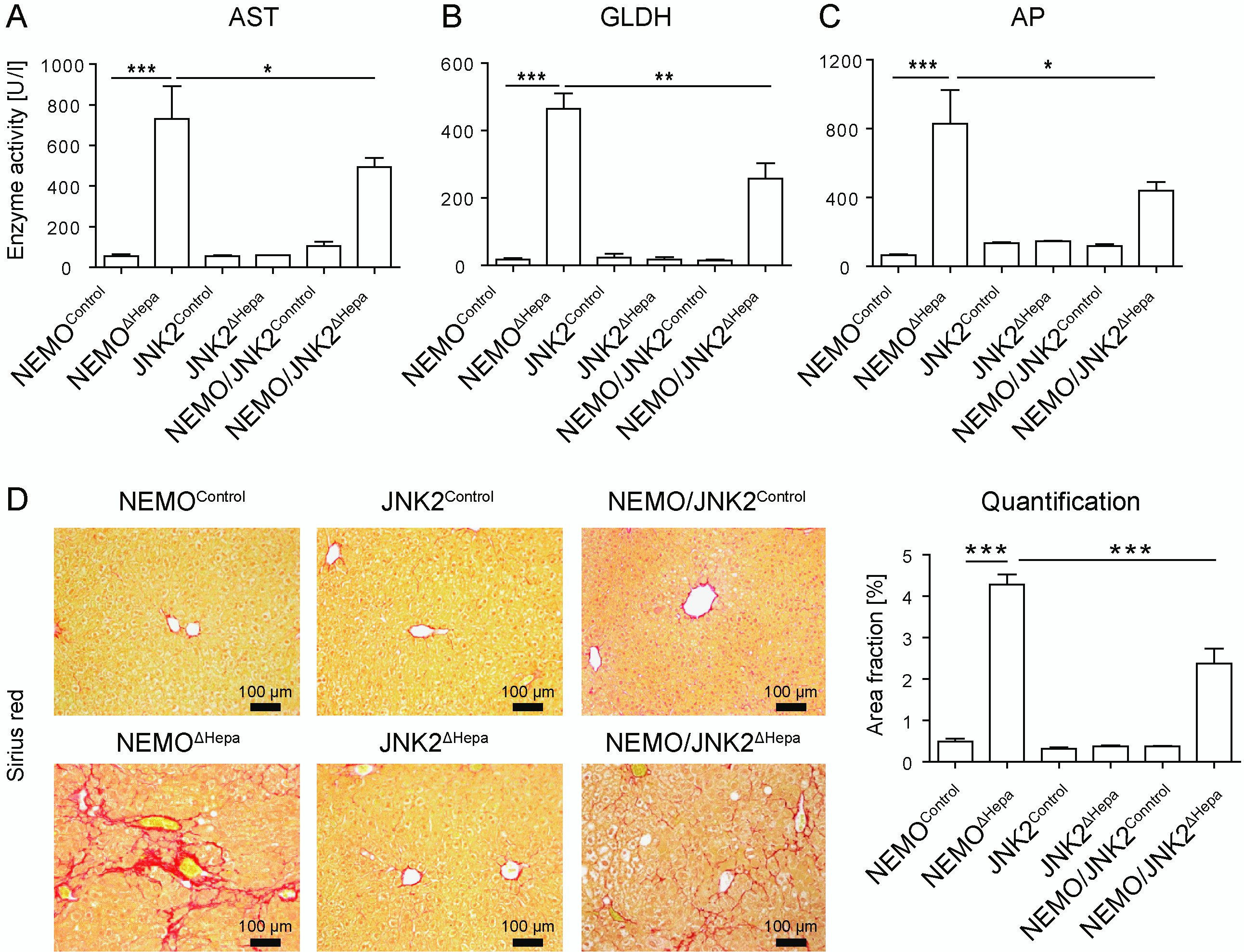

Supplement: Supplementary file 2 — Suppl. Fig. 1 [file 41419_2020_2571_MOESM2_ESM.tif]

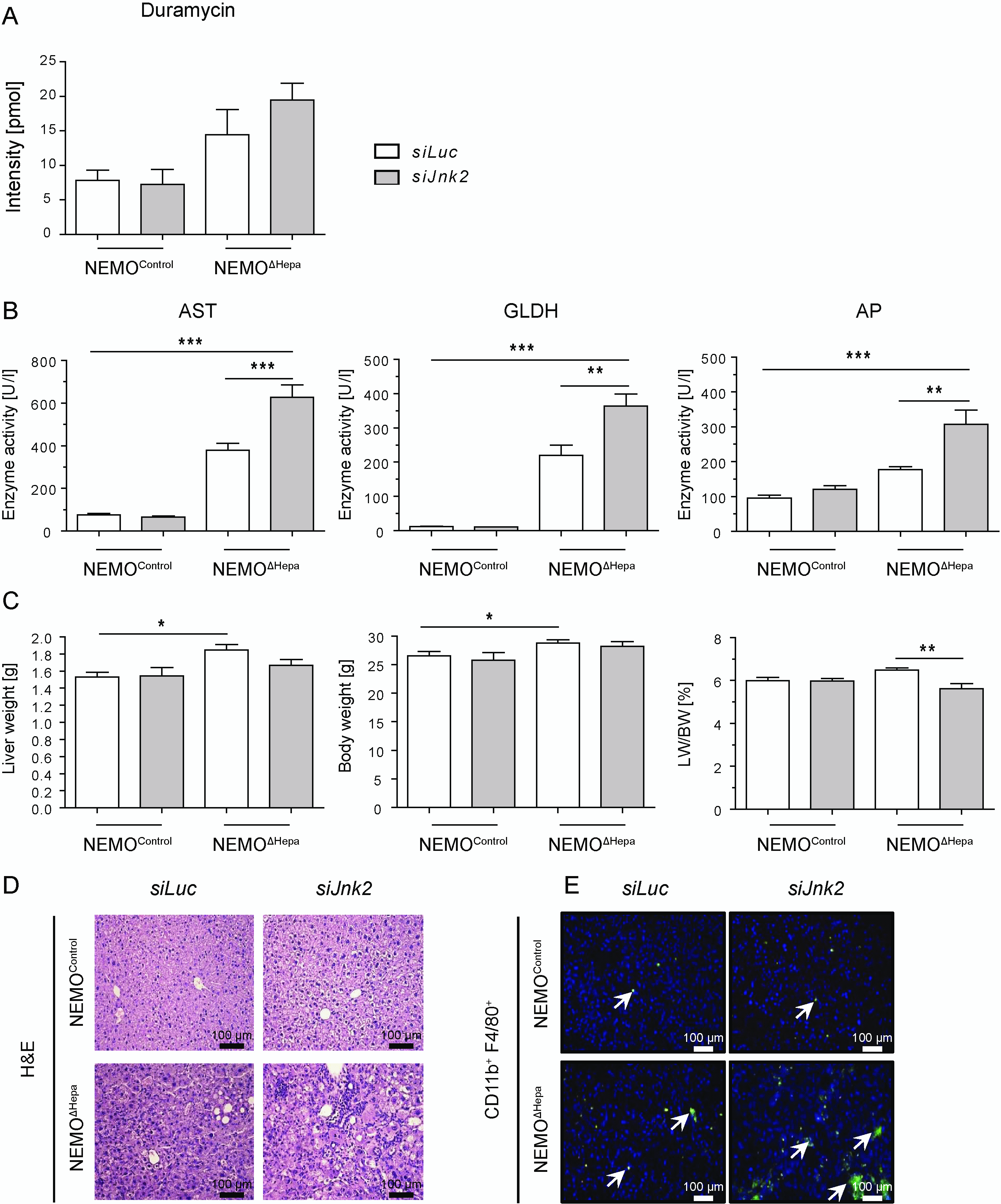

Supplement: Supplementary file 3 — Suppl. Fig. 2 [file 41419_2020_2571_MOESM3_ESM.tif]

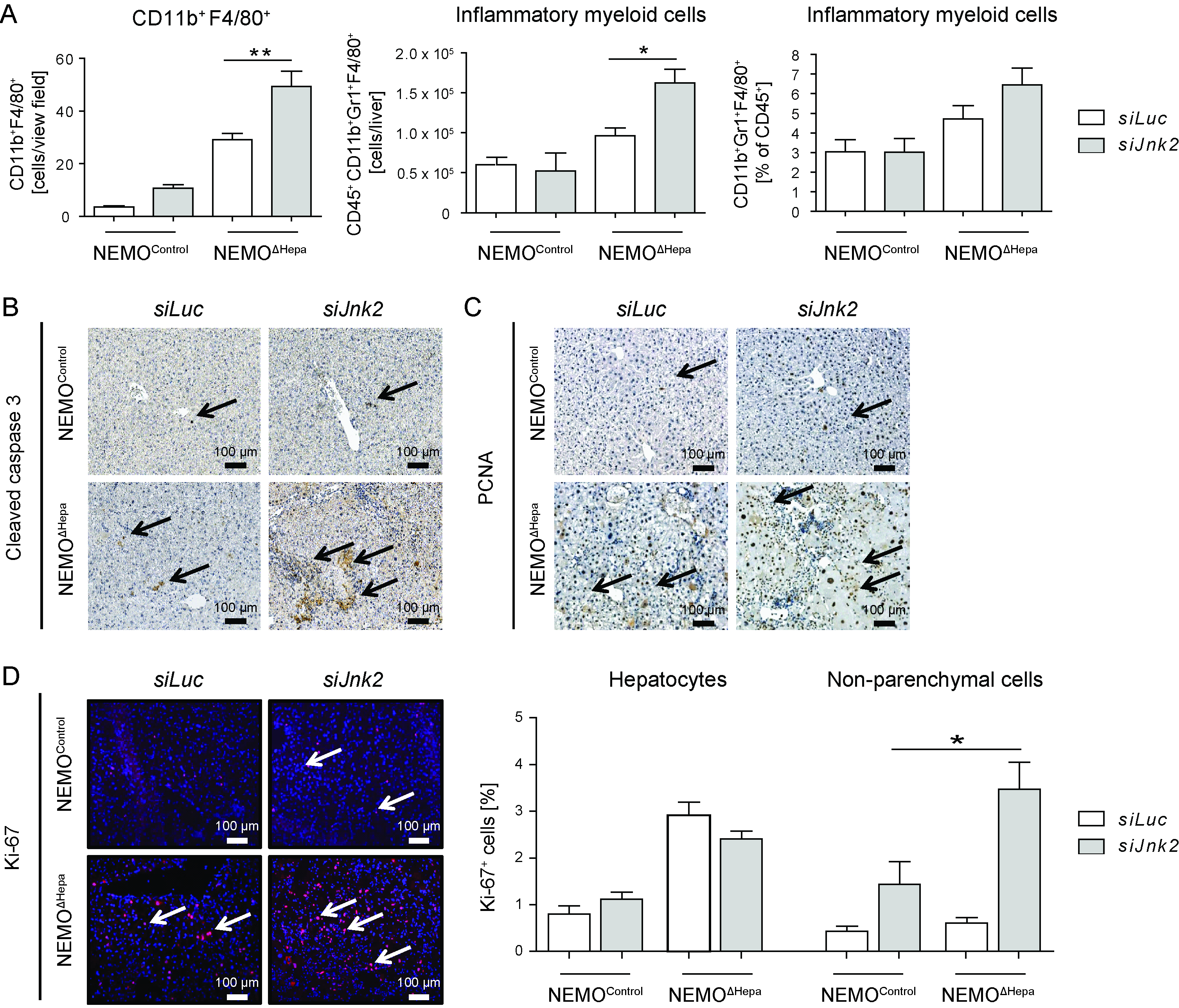

Supplement: Supplementary file 4 — Suppl. Fig. 3 [file 41419_2020_2571_MOESM4_ESM.tif]

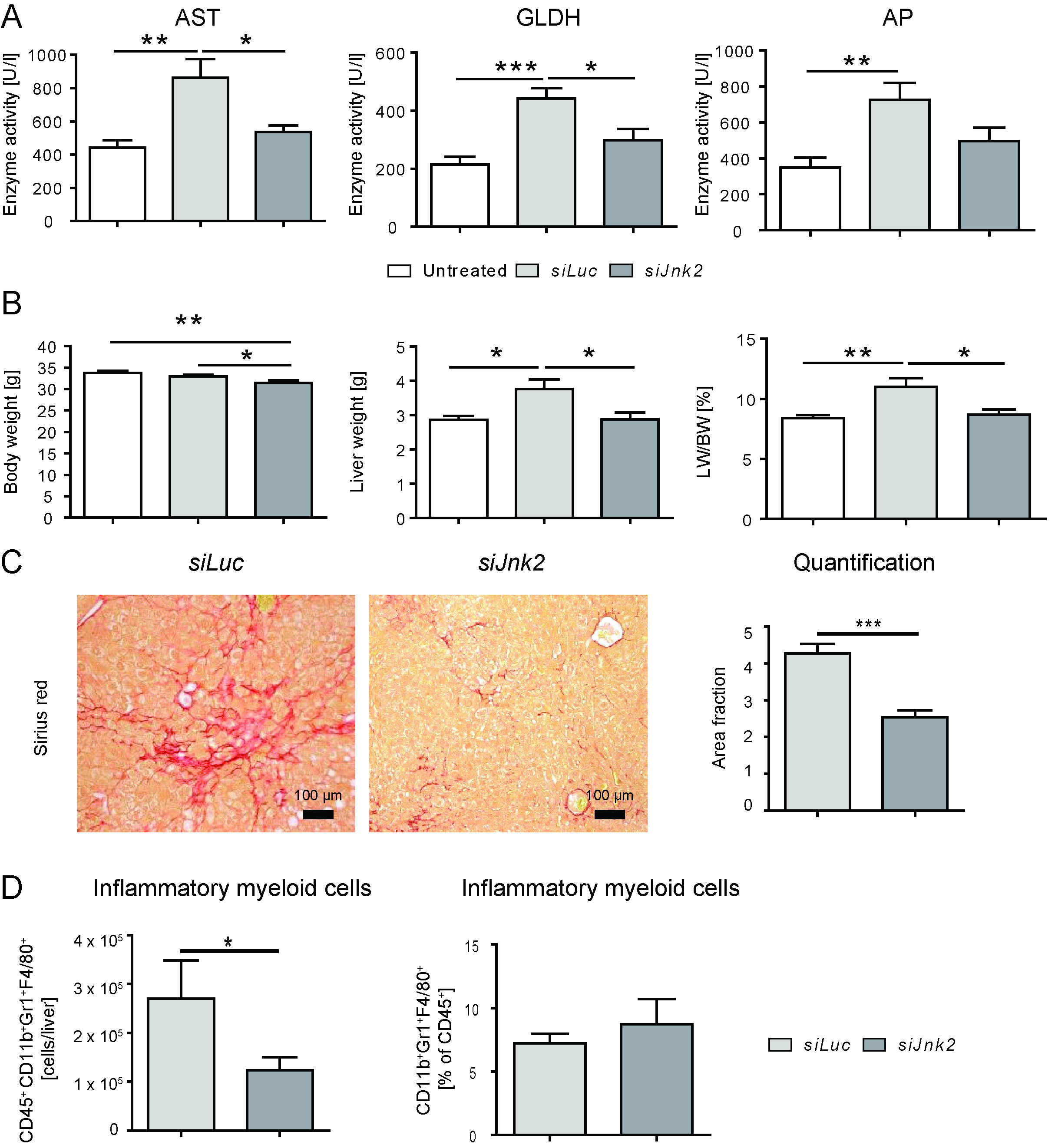

Supplement: Supplementary file 5 — Suppl. Fig. 4 [file 41419_2020_2571_MOESM5_ESM.tif]
